# The relationships of viral and protozoal co-infections to *Chlamydia pecorum* infection and chlamydiosis outcomes in northern koalas *(Phascolarctos cinereus)*

**DOI:** 10.1371/journal.ppat.1013632

**Published:** 2025-11-24

**Authors:** Yasmine S. S. Muir, Belinda R. Wright, Andrea Casteriano, Mathew S. Crowther, Amber Gillett, Mark B. Krockenberger, Damien P. Higgins

**Affiliations:** 1 Sydney School of Veterinary Science, Faculty of Science, University of Sydney, Camperdown, Sydney, New South Wales, Australia; 2 School of Life and Environmental Sciences, Faculty of Science, University of Sydney, Camperdown, Sydney, New South Wales, Australia; 3 Australia Zoo Wildlife Hospital, Beerwah, Queensland, Australia; Catalan Institute for Water Research (ICRA), SPAIN

## Abstract

Several infectious agents concurrently infect wild koalas and so, as for similar agents in other species, co-infection interactions could affect disease presentation and clinical outcomes. This study determines the frequency of circulating and mucosal *Chlamydia pecorum* infections along with phascolarctid herpesvirus (PhaHV), Koala retrovirus (KoRV), and trypanosome infections in 115 wild koalas admitted to wildlife hospitals in the Australian states of Queensland and New South Wales. *C. pecorum,* PhaHV, trypanosomes, and KoRV (endogenous subtype A and exogenous subtype D) were detected in 61.1%, 68.9%, 63.3% and 100% of the individuals sampled, respectively. The co-infection relationships identified generate hypotheses for the observed variation in disease presentations in that they resemble co-infection interactions that drive the variations in presentation and response to treatment for chlamydiosis in other species, including humans. Among koalas with chlamydiosis, PhaHV-1 mucosal shedding positively predicted euthanasia on admission, and accounting for *Trypanosome irwini* infection status improved the model quality. Additionally, in female koalas, the detection of mucosal PhaHV-1 and greater KoRV proviral *pol* loads were equal predictors of chlamydial reproductive disease. While the detection frequency of *C. pecorum,* PhaHV-1, PhaHV-2, and *T. gilletti* in circulation were low, cases with circulating *C. pecorum* and without mucosal *C. pecorum* shedding or clinical chlamydiosis were observed presenting an important consideration for future diagnostic testing. This study serves as a basis for investigating co-infection interaction pathways through mechanistic studies to determine their effect on pathogenesis of chlamydiosis, improve our understanding of host-pathogen-environment dynamics impacting the koala, and identify novel intervention and screening methods.

## Author summary

High frequency of euthanasia of koalas in care highlights a need for better understanding of the pathogenesis of infectious disease to better inform clinical interventions and population disease management. This study detected koala retrovirus (KoRV), phascolartid herpesvirus (PhaHV), *Chlamydia pecorum*, and trypanosomes in 115 northern koalas admitted to wildlife hospitals and examined the data for associations between co-infection, clinical presentation, and admission outcomes. Co-infections were common, and each infectious agent and various co-infections differed in their association with clinical manifestation of disease. Our findings indicate that PhaHV-1 and KoRV are equally important infectious agents that may contribute to the diversity of clinical presentations in the koala. Detection of circulating *C. pecorum* occurred independent of mucosal *C. pecorum* shedding or disease in some koalas. Current *C. pecorum* screening only identifies mucosal shedding, hence a small portion of Chlamydia infected koalas are not identified currently. Further investigation of the potential mechanisms by which infectious agents interact with each other, and the host, is required to understand pathogenesis and improve disease mitigation and treatment methods.

## Introduction

Koalas are separated broadly into two groups that differ geographically and genetically: the northern population (Queensland [Qld], New South Wales [NSW], & Australian Capital Territory) and the southern population (Victoria & South Australia) [1,2]. In 2022, the northern koala population was listed as endangered under Australian Commonwealth legislation [3]. In NSW, between 2020–2021, 46.8% of rescued koalas died or were euthanised [4]; a rate that is 3.1% and 5.6% greater than those recorded in 2019–2020 and 2018–2019, respectively [4]. Although the catastrophic ‘Black Summer’ bushfires of 2019–2020 likely contributed to increases in admissions during that period, on average 71% of koalas admitted for chlamydiosis die and 25% are released [4], suggesting room for improvement in clinical management of disease in koalas. In many regions of Australia, non-traumatic disease is the leading cause for koala admission to wildlife hospitals [5], with chlamydiosis due to *C. pecorum* the leading cause in NSW (2018–2021) [4]. This obligate intracellular bacterium is present in most mainland koala populations and affected animals classically present with conjunctivitis and/or ‘wet bottom’, a urine-stained rump from incontinence due to cystitis. Both presentations range from mild, acute inflammation to severe and chronic proliferative or fibrotic disease in which the structural tissue damage can result in renal failure, blindness, secondary infection, death or infertility. Infertility is a great concern for population viability, and in females is due to inflammation and associated scarring of the uterus and uterine tubes (pyometra, endometriosis, ovarian bursal cyst formation, obstructive fibrosis and tubal defects) and in males inflammation of the epididymis, prostate or testes, resulting in sperm reduction, damage & abnormal morphology [6–11].

Due to the high probability of infertility and in some cases poor quality of life [12], the presence of ovarian bursal cysts is one criterion for euthanasia supported by the Code of Practice for the Care of Sick, Injured or Orphaned Protected Animals in Queensland and Code of Practice for Injured, Sick and Orphaned Koalas in New South Wales (NSW [13,14]. In other species, the pathogenesis of chlamydiosis is complex, comprising intestinal, urogenital, ocular and systemic infection and interactions with other viruses and bacteria, as well as a range of stressors [15–23]. Chlamydial infection in humans and animals can also be sub-clinical [24–27], and has been reported as such in the koala [28,29]. Overall, the mechanisms behind clinical variation in chlamydiosis outcomes are poorly understood.

In humans and other animals, co-infections are common and influence the severity and progression of chlamydial infection through various mechanisms involving different host, pathogen, and environmental factors [30,17]. As observed in experimental mouse models of *C. trachomatis* and other bacterial and viral co-infection, specific effects of co-infections on *C. pecorum* activity and chlamydiosis in the koala could be highly dependent on the particular infectious agents involved, the timing and sequence of infections, and the host’s immune status [17,22]. The interplay between different agents in a co-infection scenario is complex, causing alterations to pathogenesis through mechanisms such as immunomodulation, competition for resources, synergistic effects, antagonistic effects, recurrence and reactivation, and disease modulation [30]. Variations in clinical disease presentations, treatment response, and pathogen detection highlight key gaps in our understanding of pathogenesis and the roles of non-chlamydial infectious agents in driving host-pathogen-co-pathogen interactions. The list of infectious agents of putative significance to the pathogenesis of chlamydiosis in koalas is growing and now includes endogenous and exogenous Koala retrovirus (KoRV) [31–33], phascolarctid gamma-herpesvirus (PhaHV) [34,35], and trypanosomes [36]. In the northern koala population, KoRV subtype A is endogenous and ubiquitous, while exogenous subtypes B-M are locally prevalent [37–42]. Various KoRV-associated parameters have been associated with chlamydiosis [37,43,44,40,45], and it is postulated that these associations exist due to the KoRV immunosuppressive domain (ISD) [46,47,31], circular interaction with oxidative stress and inflammation [48], amplification of innate anti-viral immune defences [49], or insertion adjacent to immune genes as observed in retroviruses in humans [50,51].

Two types of gammaherpesvirus have been identified in the koala so far: PhaHV-1 [52] and PhaHV-2 [53]. PhaHV-1 shedding increases with age and PhaHV-2 shedding is more common in euthanased koalas with poorer body condition [54,55]. A number of possible pathogen interactions have already been noted. PhaHV-1 and exogenous KoRV A infection is associated with uterine/ovarian bursal cysts and testicular malformation, reduced female fertility, urinary incontinence, and the co-detection of *C. pecorum* in South Australian (SA) koalas [35]. In humans, herpes simplex virus Type-2 and *Chlamydia trachomatis* have a dynamic relationship that, depending on which is the primary infection, can significantly alter pathogenesis [56].

Of the four species of trypanosome known to infect koalas (*T. copemani, T. Irwini, T. gilletti, and T. vegrandis)*, *T. gilletti* has been suggested to exacerbate anaemia and body condition loss in northern populations [57,36]. More specifically, koalas with signs of chlamydiosis that were also infected with *T. gilletti* had significantly lower blood packed cell volumes and body condition scores compared to non-trypanosome infected diseased koalas [36]. These association studies, whilst not yet providing information on causation, are an important and necessary step that guide further focused analyses into potential causal relationships between co-infection risk factors and health outcomes.

With koala populations declining across their northern range, we must establish which infectious agents are likely to impact disease mitigation, prevention, and conservation efforts. Before co-infection associated modulation of the koalas’ host response can be explored, the types of co-pathogen interactions and their association with clinical outcomes must be established. To this end, this study utilises gold-standard and novel methods to detect and quantify parameters for four key groups of infectious agents; *C. pecorum*, KoRV, PhaHV-1 & -2, and trypanosomes to: (1) determine the potential for the NanoString RNA hybridization assay to detect koala pathogens in circulation, (2) determine the detection frequency of these infectious agents in a sample population of 115 wild northern koalas with diverse clinical presentations, demographics, and geographical origins, admitted to three wildlife hospitals within NSW and Qld, (3) determine whether co-infections are preferentially associated with *C. pecorum* infection in koalas on admission, and (3) assess the relationships of co-infections with clinical chlamydiosis. Future directions are suggested to further explore the relationship between co-infections to identify pathological mechanisms which may be involved in persistent/recurring infections and the presentation of clinical signs and poor responses to chlamydiosis treatment necessitating euthanasia.

## Results

### Frequency of C. pecorum detection

In the current study, detection of *C. pecorum* mRNA from buffy coats identified cases potentially infected by *C. pecorum* that were not identified using DNA detection at mucosal sites alone. Of 93 koalas with conclusive results for all *C. pecorum* detection parameters, 61.1% (51/93) were positive for at least one *C. pecorum* target (Table 1). In these *C. pecorum* positive koalas, mucosal *C. pecorum* was most frequently detected (86.3%, 44/51), followed by combined mucosal and circulating *C. pecorum* (7.8%, 4/51), then by circulating *C. pecorum* only (5.8%, 3/51). Nine mucosal and 17 buffy coat samples were excluded from analysis due to either missing or poor-quality extracts.

**Table 1:** Detection frequency of infectious agents and specific targets on admission.

*Infectious agent*	*Sample Type*	*Target*	*N samples tested*	Positive count	Positive frequency (%)
*C. pecorum*	*Mucosal*	*Ocular*	110	25	22.7
		*Urogenital*	108	56	51.8
		**Total Mucosal** ^**1**^	106	60	56.5
	*Circulating*	*G_0573*	98	2	2.0
		*Hsp60*	98	4	4.1
		*OmpA*	98	1	1.0
		*Pgp3*	98	1	1.0
		**Total Circulating** ^**2**^	98	8	8.2
PhaHV	*Mucosal*	PhaHV-1	112	66	58.9
		PhaHV-2	112	24	21.4
		**Total Mucosal** ^**1**^	112	71	63.4
	*Circulating*	PhaHV-1	98	2	2.0
		PhaHV-2	98	4	4.1
		**Total Circulating** ^**2**^	98	5	5.0
KoRV	*Circulating*	KoRV *pol* DNA	112	112	100
		KoRV *pol* cDNA	113	113	100
		KoRV *pol* mRNA	98	98	100
		KoRV *env* A	98	98	100
		KoRV *env* B	98	33	34.7
		KoRV env D	98	98	100
		KoRV *env* CKS17	98	98	100
*Trypanosomes*	*Circulating*	*T. irwini*	98	59	60.2
		*T. copemani*	98	27	27.6
		*T. gilletti*	98	3	3.1
		**Total Circulating** ^**2**^	98	62	63.3

1. Grouping all positive results for infectious agent at mucosal sites: *C. pecorum* at UGT or ocular sites, and PhaHV-1 or PhaHV-2 at oropharyngeal site.

2. Grouping all positive results for infectious agent gene transcription to represent circulating presence: *C. pecorum = G_0573, Hsp60, OmpA and/or PgP3,* PhaHV = PhaHV-1 and/or PhaHV-2, Trypanosomes = *T. copemani, T. gilletti,* and/or *T.irwini.*

### Frequency of PhaHV-1 & PhaHV-2 detection

PhaHV DNA and/or mRNA was detected in 68.9% (73/106) of koalas in the study cohort. Irrespective of detection site, PhaHV-1 and PhaHV-2 infection was identified in 62.1% (64/103) and 26.7% (28/105) of koalas, respectively. PhaHV detection largely consisted of mucosal PhaHV-1 or combined detection of mucosal PhaHV-1 and PhaHV-2 (Table 2). Overall, there were a total of 95/115 samples with available results for all PhaHV targets (Table 2). Single type PhaHV infection was more common than combined PhaHV-1 and PhaHV-2 co-infection; 75.8% (47/62) vs 24.2% (15/62), respectively (Table 2). Detection of PhaHV-1 and PhaHV-2 in circulation was rare: 2.1% (2/95) and 4.2% (4/95), respectively (Fig A in S1 Text). Two cases with circulating PhaHV-2 did not have detectable mucosal shedding. PhaHV-2 mucosal detection was significantly associated with geographic origin (p < 0.001). The odds of a koala shedding mucosal PhaHV-2 was 6.33 times greater if they originated from NSW compared to Qld (12/24, 50%; vs 12/78, 15.4%, c^2^ = 13.2, df = 1, p < 0.001; Odds ratio = 6.33, 95%CI = 2.3-17.9). It should be highlighted that 75% (9/12) of PhaHV-2 positive koalas from NSW originated from Port Macquarie, while koalas from Port Macquarie only represented 45% (12/25) of those from NSW in this study, suggesting overrepresentation in that area.

**Table 2:** Frequency of detection of mucosal and circulating PhaHV -1 & -2 in koalas with complete results for all targets.

PhaHV Infection Combinations	Count	%
**PhaHV1 Mucosal**	40	42.1
**PhaHV2 Mucosal**	4	4.2
**PhaHV2 Circulating**	2	2.1
**PhaHV1 Mucosal x PhaHV2 Mucosal**	13	13.6
**PhaHV1 Mucosal x PhaHV1 Circulating**	1	1.1
**PhaHV1 Mucosal x PhaHV2 Circulating**	1	1.1
**PhaHV1 Mucosal x PhaHV1 Circulating x PhaHV2 Circulating**	1	1.1
**None**	33	34.7
**Total**	95	100

### Frequency of KoRV detection

**Fig 1:**
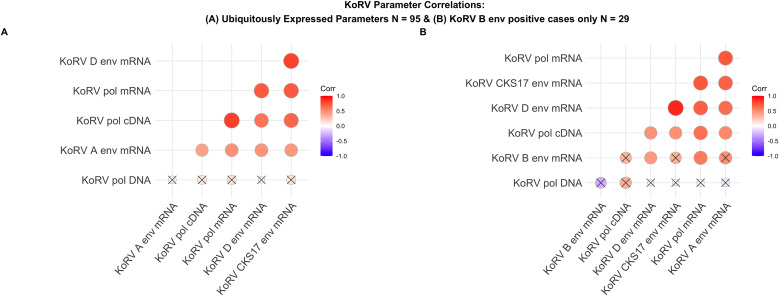
Pearson’s correlation matrices of KoRV markers. (A) Depicts correlations between parameters without KoRV B results using 95 observations. (B) Correlations between all markers, inclusive of KoRV B, using 29 observations. Correlations marked X are insignificant (p > 0.05).

All KoRV parameters, apart from KoRV-B *env* mRNA (detection frequency 33/98, 33.7%), were detected ubiquitously and were quantifiable in the analysed sample population (Table 1 and Fig 1). All KoRV parameters were non-normally distributed and were Log_10_ transformed to assess Spearman’s correlations. Strong and significant correlations existed between most KoRV *pol* cDNA (qPCR) and mRNA (Nanostring) parameters (Fig 1). Therefore, to avoid effects of multicollinearity in downstream models, KoRV *pol* cDNA/mL was used as a representative for KoRV transcription in further analysis, due to its greater sample size. KoRV *pol* proviral DNA copies/mL did not correlate with cDNA or mRNA parameters and so it was retained as an independent predictor in further analysis.

### Frequency of Trypanosome detection

Trypanosome mRNA was detected in 63.3% of analysed buffy coat samples (62/98) with gene transcription for more than one species detected in 40.3% of those (25/62). *T. irwini* was the most prevalent trypanosome infection, followed by *T. copemani*. *T. gilletti* was the least prevalent (3/98) and 100% of *T. gilletti* detection occurred alongside either *T. copemani* or *T. irwini.* Similarly, 88.9% of samples with *T. copemani* mRNA also had evidence of *T. irwini* mRNA. Due to low frequency of detection for *T.* gilletti (S2 Fig in S1 Text), only *T. copemani* and *T. irwini* were assessed further.

### Frequency of chlamydial co-infections

**Fig 2:**
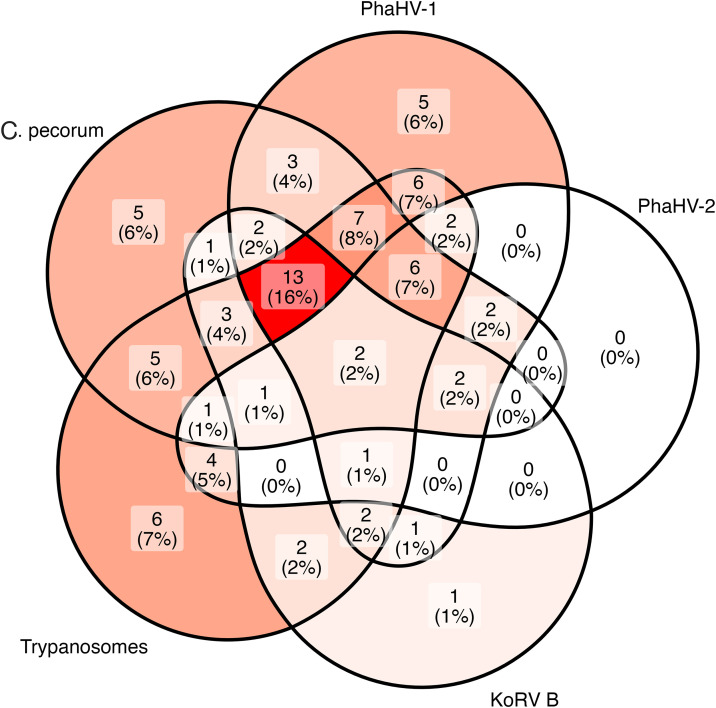
Euler Venn diagram of complete pathogen detection status. *Euler Venn diagram demonstrating the full pathogen detection results for 92 koalas with complete results for all targets. Positive detection of different markers representing the same pathogen were combined so that each case had one result per pathogen:* C. pecorum *includes detection at mucosal sites and/or transcription of genes* CpecG_0573, Cpec_hsp60, ompA, or pGP3 *in circulation, PhaHV-1 and PhaHV-2 includes detection of each respective type at mucosal sites or in circulation, Trypanosomes include detection of any of the three species,* T. copemani, T. gilletti, *and/or* T. irwini *in circulation. KoRV B is depicted independently from other KoRV targets as it was not ubiquitously detected. All 92 koalas were positive for KoRV* pol, *KoRV A* env, *KoRV D* env, *and KoRV CKS17* env *transcription in circulation. In total, 23/115 koalas in the whole sample population had missing/inconclusive results for any one pathogen and were exluded.*

The detection frequencies of most infectious agents assessed were high within the examined population and, as a result, the frequency of co-infection among *Chlamydia*-infected koalas was also high (Fig 2). Of the 66 koalas in which mucosal and/or circulating *C. pecorum* was detected (61.1%), only 5 cases (6%) had *C. pecorum* infection alone (excepting the ubiquitously expressed KoRV parameters, Fig 2). The odds that a *C. pecorum* infected koala was co-infected with mucosal or circulating PhaHV -1 and/or PhaHV -2 was 3.1 times the odds of a *C. pecorum* negative koala (49/63, 77.8%; vs 21/40, 52.5%, χ^2^ = 6.06, df = 1, p = 0.014; Odds ratio = 3.12, 95%CI = 1.32-7.55, p = 0.009). Specifically, koalas with *C. pecorum* infection had 2.8 times and 2.9 times the odds of shedding PhaHV-1 or PhaHV-2, respectively: 44/65, 67.7%; vs 17/40, 42.5%; χ^2^ = 5.46, df = 1, p = 0.019; Odds ratio = 2.79, 95%CI = 1.24-6.45, p = 0.012, and 19/66, 28.7%; vs 5/42, 11.9%; χ^2^ = 3.31, df = 1, p = 0.068; Odds ratio = 2.91, 95%CI = 1.04-9.64, p = 0.04. Mucosal loads of *C. pecorum* DNA were not significantly correlated with either PhaHV-1 or PhaHV-2 DNA loads (N = 38, Cor = 0.08, p > 0.05; N = 16, r = 0.05, p > 0.05). When co-infection combinations were assessed in koalas with results for all infectious agent targets (N = 92), the most common infection combination included *C. pecorum*, PhaHV-1, KoRV B and trypanosome tri-infection irrespective of detection site or trypanosome species (13/92, Fig 2).

### General Linear Models: The relationships between co-infections and disease outcomes

#### Clinical characteristics of tested koalas.

**Fig 3:**
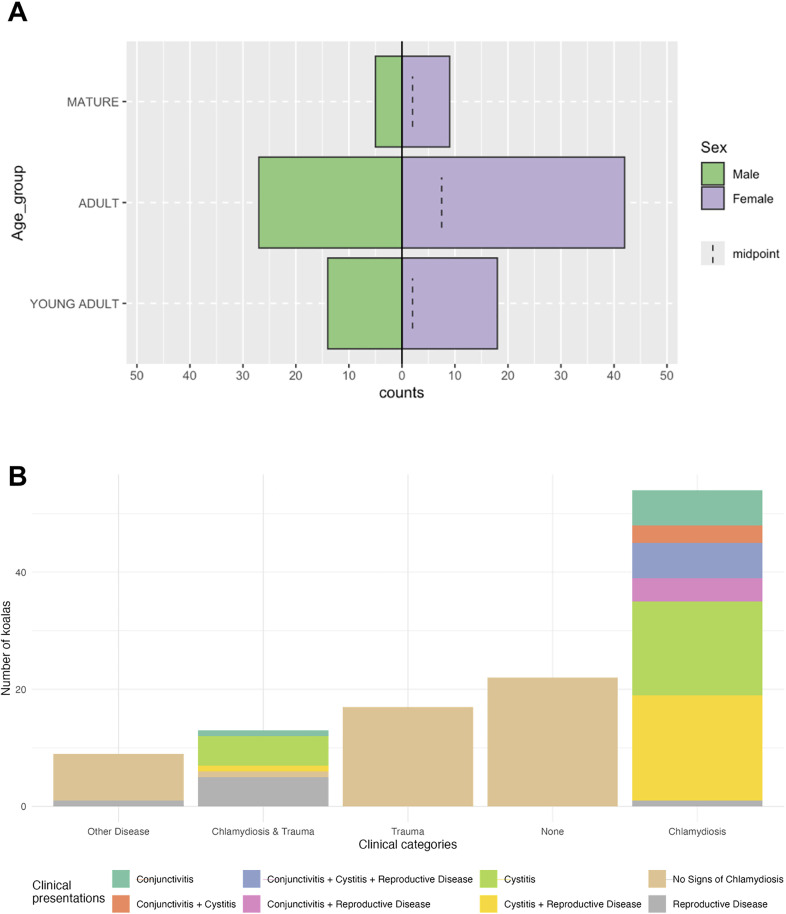
Distribution of age, sex, and clinical presentations. Graphical summaries of demongraphic and clinical status. (A) Pyramid plot showing counts of koalas within each age group according to sex. (B) Bar plot of the clinical allocation count for 115 koalas admitted to hospital including the proportion of specific chlamydiosis-clinical signs detected in cases.

Of the 115 koalas sampled on admission to hospitals, 61% presented with clinical signs of chlamydiosis including any one or combination of cystitis, conjunctivitis and reproductive disease (Fig 3B). The remaining koalas presented with trauma (n = 17), disease other than chlamydiosis (n = 8), and no clinical abnormalities (n = 20). In total, 71 koalas (62% of admissions) were retained for rehabilitation and the remainder were euthanised following clinical assessment. Of the euthanised koalas, 84% (N = 37) were classed as ‘untreatable chlamydiosis’. Female koalas represented 60% of the sampled population (Fig 3A). Over half of female koalas presented with evidence of reproductive disease on admission with or without other signs of chlamydiosis. Reproductive disease was the leading cause for euthanasia in the sample population (45.5%).

The distribution of age groups and sex were unequal within the sampled cohort (Fig 3A). Females were more frequently represented by the following categories: Chlamydiosis (χ^2^ = 7.94, df = 1, p < 0.01), non-treatable chlamydiosis (χ^2^ = 12.96, df = 1, p < 0.001), cystitis (χ^2^ = 5.51, df = 1, p < 0.05), and reproductive disease (χ^2^ = 32.55, df = 1, p < 0.001). Significantly fewer young-adult koalas were shedding mucosal *C. pecorum* in comparison to adults (χ^2 ^= 8.42, df = 2, adj. p < 0.05) and mucosal PhaHV-1 in comparison to adults (χ^2 ^= 16.55, df = 2, adj. p < 0.001) and aged koalas (χ^2 ^= 16.55, df = 2, adj. p < 0.01). Examination for male reproductive disease was not conducted in this study, so modelling for reproductive disease was only performed on the female subset.

#### *C. pecorum* mucosal shedding.

The selected “best model” explaining the differences in likelihood of shedding *C. pecorum* at mucosal sites (urogenital or ocular) included positive relationships with female koalas, detection of KoRV-B *env,* mucosal PhaHV-2 shedding, and with KoRV *pol* transcription (Table 3). Although the top model included *T. copemani* as a predictor, the relative importance < 0.50 and in the next model, which didn’t include *T. copemani,* the penalisation of AIC_c_ and w_i_ was considered minor (Δ_*i*_ = 0.20, and change in w_i_ = 0.1). After sex, KoRV-B *env* detection was considered the most important pathogen predictor variable, followed by PhaHV-2 mucosal infection, then KoRV *pol* transcription. Of note, the 95% confidence intervals (CIs) for the three pathogen predictors included 1, indicating that while these variables showed positive associations of relative importance with *C. pecorum* mucosal shedding, the increases in odds were not statistically significant in this sample.

**Table 3:** Best model selections using Akaike Information Criterion corrected for small sample sizes (AICc) and model averaging. Models were generated from a global model and ranked by AICc values. Model averaging was applied using Akaike weights.

Best Model	Number of observations	Number of parameters	AICc	Delta AICc (Δ*i*)	Akaike weight (*wi*)	Coeff	SE	95% CI		Relative importance
								2.5%	97.5%	
*C. pecorum* mucosal shedding	87	5	114.96	0.20	0.9					
Sex: Female						1.04	0.48	0.11	2.03	1
Circulating *KoRV B*						1.02	0.55	-0.04	2.14	0.87
Mucosal PhaHV-2 shedding						1.02	0.61	-0.13	2.28	0.62
Log_10_ KoRV *pol* cDNA/ml						0.79	0.46	-0.09	1.75	0.60
Chlamydiosis	87	3	73.44	0.00	0.21					
*C. pecorum* mucosal shedding						3.39	0.67	2.17	4.87	1
Sex: Female						1.63	0.67	0.37	3.05	1
Mucosal PhaHV-1 shedding						1.24	0.65	0.004	2.59	0.91
Untreatable chlamydiosis	48	3	51.34	0.00	0.17					
Mucosal PhaHV-1 shedding						2.41	0.86	0.84	4.27	1
Sex: Female						2.73	0.90	1.10	4.72	1
Circulating *T. irwini*						-1.42	0.89	-3.36	0.21	0.67
Reproductive disease	50	3	54.40	0.00	0.45					
*C. pecorum* mucosal shedding						2.49	0.79	1.04	4.24	1
Mucosal PhaHV-1 shedding						1.71	0.80	0.23	3.45	1
Log_10_ KoRV *pol* DNA/ml						3.49	1.87	0.91	8.97	1

Each model presented AIC_c_ values, Akaike weights (*wi*) and delta AIC_c_ differences (Δ_i_). Standardised model-averaged coefficients (Coeff) weighted unconditional standard errors (SE), 95% confidence intervals (95% CI) and relative importance are provided for each independent variable in the best-supported models. Relative importance values below 0.5 are shown in red.

#### Chlamydiosis.

The best model explaining the differences in the likelihood of koalas presenting with chlamydiosis on admission included positive relationships with sex (female), mucosal *C. pecorum* shedding, and mucosal PhaHV-1 shedding. This model had the lowest AIC_c_ and included all predictors with relative importance > 0.50. *C. pecorum* mucosal shedding and sex (female) were of equal greatest importance to the prediction of chlamydiosis. This was followed by mucosal PhaHV-1 shedding.

#### Untreatable chlamydiosis.

The best model explaining differences in the probability of koalas with chlamydiosis requiring euthanasia on admission included a positive relationship with sex (female) and PhaHV-1 mucosal shedding, and a negative relationship with circulating *T. irwini* infection. However, the 95% CI for circulating *T. irwini* included 1, indicating that while this variable showed relatively important negative association with koalas with chlamydiosis being untreatable, the decrease in odds was not statistically significant in this sample.

#### Reproductive disease in females.

The best model explaining differences in the probability of female koalas presenting with reproductive abnormalities on admission included positive relationships with mucosal *C. pecorum* shedding, mucosal PhaHV-1 shedding, and KoRV *pol* proviral loads. This model had superior AIC_c_ and w_i_ scores and included all predictors with relative importance > 0.50. In fact, all three predictors demonstrated equally high levels of relative importance to the prediction of reproductive disease in females.

## Discussion

**Fig 4:**
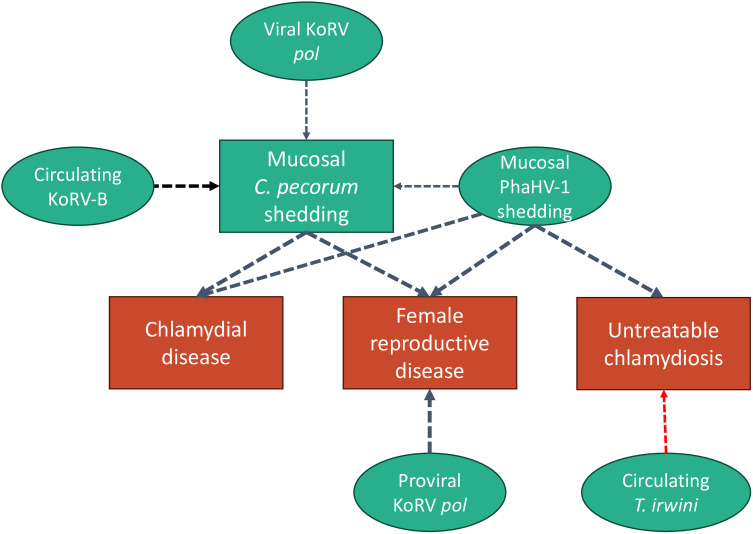
Relative Importance (RI) Network Plot demonstrating the degree of importance of infectious predictor variables on clinical outcome variables. All variables included as predictors in best models are represented in green ovals, and outcome variables in red and green squares. The arrows indicate the direction and positivity (black) or negativity (red) of the interaction. While these arrows are unidirectional to clearly define the model design, the independent and dependent variables, this study does not denote causation and bidirectionality may exist. Only predictors with RI > 0.5 were retained in best model fits and are displayed here. The thickness of the dotted arrows represents the magnitude of RI (0.5-1).

The findings of this study demonstrate a need to reassess the current understanding of host-pathogen-environment relationships involved in chlamydiosis of koalas. This first collective examination of key infectious agents in northern koalas showed that PhaHV-1 and KoRV were fairly equal in terms of importance to the prediction of chlamydiosis outcomes. Of all assessed pathogen targets, the relationship between clinical chlamydiosis and *C. pecorum* infection appears to be intertwined with PhaHV-1 shedding, KoRV transcription and replication, and to a lesser extent, *Trypanosome irwini* infection (Fig 4). Although the detection of *C. pecorum* and PhaHV-1 and -2 were greater at mucosal sites, the detection of circulating pathogen genes (*CpecG0573, Cpec_hsp60, ompA, Pgp3, PhaHV-1 dpol, PhaHV-2 dpol,* and *Tgilletti18S),* albeit at low prevalence, suggests that further studies are required to determine their significance to pathogenesis. In particular, the finding of circulating *C. pecorum* gene transcription in koalas without detectable mucosal *C. pecorum* shedding indicates that diagnostic testing of circulatory cell samples should continue to be validated. The strong associations found here between PhaHV-1, *C. pecorum,* and chlamydiosis mirror those identified in humans and mouse models and the pathogenesis of these relationships is worth further exploration. Finally, given the combination of expected (KoRV B and viral KoRV *pol)* and unexpected (proviral KoRV *pol)* associations of KoRV with mucosal *C. pecorum* and chlamydiosis, we discuss the complexities of KoRV’s significance in this condition.

The incorporation of all key koala infectious agents into a single analysis in this study demonstrated equal importance of PhaHV-1 and KoRV to koala chlamydiosis and a high frequency of co-infection. Previously, the relationships of these agents with disease in northern koalas have been studied separately, resulting in an incomplete understanding of the relative importance of co-infecting agents and their interrelationships. Although a study of PhaHV-1, -2, *C. pecorum* and KoRV co-infections was conducted in southern koalas [35], direct comparison of our results is limited due to the differences host genetic backgrounds and the differing KoRV host-pathogen relationships [38,58,59,60]. Here, the inclusion of *Trypanosome* detection revealed that the most common co-infection included *C. pecorum*, PhaHV-1, KoRV (including *env* subtypes KoRV A, B, & D), and at least one *Trypanosome* species. Similarly to Vaz et al. [35], older koalas and those with *C. pecorum* had high odds of also being co-infected with either PhaHV-1 and/or PhaHV-2. As expected, and corroborating Vaz et al. [35], PhaHV-1 shedding was a strong predictor of *C. pecorum* shedding and chlamydiosis, but, in contrast to Vaz et al. [35], PhaHV-1 shedding and KoRV proviral *pol* loads were equally important indicators of reproductive disease in females.

Collectively, our results suggest that to better understand chlamydiosis in northern koalas both KoRV and PhaHV should be examined alongside *C. pecorum* to account for possible interactions. While co-infection is yet to be investigated as a mechanism driving the variations in the pathogenesis of chlamydiosis in koalas, evidence of co-infection relationships exists in humans, mouse models, and other animals infected with other *Chlamydia spp.* [61,62,63]. Considering similar relationships in chlamydial disease of humans, association of herpesviral and chlamydial co-infection with chlamydial disease outcomes in koalas in this study suggests that these co-infection interactions should be further investigated. In this study, over 70% of *C. pecorum* positive koalas were coinfected with either PhaHV-1 and/or PhaHV-2. Supporting a study in southern Australian koalas [35], mucosal PhaHV-1 was strongly associated with *C. pecorum* infection and clinical disease. Contrasting to Vaz et al. [35], mucosal PhaHV-2 was not strongly associated with the detection of *C. pecorum* (circulating and/or mucosal) or clinical disease, but this was likely impacted by low detection frequencies in this study.

Although our Relative Importance network (Fig 4) reflects these interactions with a unidirectional arrow, we emphasise that this study does not denote causation. Given the propensity for herpesviruses to be reactivated with tissue and DNA damage and immunosuppression in humans [64–67] and other animals [68–70], the relationship between PhaHV-1, *C. pecorum* infection and chlamydiosis may be bi-directional. As in koalas, the pathogenesis of reproductive disease and other fibrotic conditions associated with chronic chlamydiosis of humans has not been determined [71]. However, urogenital microbiome and virome composition and, in particular, chlamydial and herpes viral co-infection in people, are areas of significant interest [72–74].

Given the relationship between PhaHV, *C. pecorum,* and disease severity in this study, these two infectious agents appear likely to be synergistic, but outcomes could depend on which pathogen infected first [30]. *C. trachomatis* and HSV-2, are currently the leading sexually transmitted pathogens in humans globally [16,56]. Disease induced by HSV-2 infection comprises genital ulcers, dysuria, cervicitis, and inguinal lymphadenopathy [75]. Early epidemiological observations suggested that women positive for both pathogens experience more severe outcomes than are typically experienced during single infections with either pathogen [76,77]. These observations were supported more recently by studies assessing the incidence of disease in mixed and single *Chlamydia* sp. infection [78] and the disease outcomes from HSV-2 and *Chlamydia muridarum* single and mixed infections in mouse models [79]. Studies have also demonstrated an antagonistic relationship between HSV-2 and *C. trachomatis*, with HSV entry into host cells causing down regulation of cell-junction protein nectin-1, on which chlamydial development relies [80,56]]. In murine models, HSV-viral recovery and disease was reduced in *C. trachomatis* pre-infected subjects and this protection ceased when viable *C. trachomatis* was removed (either naturally or through antibiotic treatment) [80,56]). The interplay between herpesvirus and chlamydial activity and latency/persistence is clearly complex but strong associations between these two infectious agents and various clinical outcomes warrants further investigation.

Although trypanosome and *Chlamydia spp.* co-infection dynamics are yet to be investigated in other species, trypanosomes can supress the establishment of infection by co-pathogens [81–84] and produce trans-sialidase enzymes, which can impact *C. pneumoniae* viability [85]. Previously, *T. gilletti* was associated with anaemia and poor body conditions in koalas with chlamydiosis [36], but *T. gilletti* was infrequently detected in this study (3.1%) and so was not assessed in general linear models. Although the opposing relationship between *T. irwini* infection and chlamydiosis severity was not significant, accounting for *T. irwini* detection improved the predictive quality of the model. Hence, our study suggests either the possibility of trypanosome species specific interactions with *C. pecorum* or an indirect effect of *T. irwini* infection on chlamydiosis pathogenesis. Transcriptomic analysis of mucosal and buffy coat samples from koalas with no infections, single infections (Trypanosome spp., PhaHV-1, or PhaHV-2, or *C. pecorum*), and co-infections should be utilised to: (1) compare gene expression between sites, (2) identify any differences in pathogen specific persistence/latency associated gene expression, (3) explore the likely mechanisms that drive persistence/latency, and (4) generate a detection method which assesses appropriate gene targets that are indicative of the various phases of activity in PhaHV and *C. pecorum*.

The differing relationships between chlamydiosis outcomes and viral and proviral KoRV *pol* loads in the current study could be a temporal effect. The strong correlations between KoRV A, KoRV B (in positive cases), KoRV D, KoRV CKS17 and KoRV *pol* transcription is consistent with previous studies demonstrating that increased exogenous subtype transcription is a significant contributor to increased viral KoRV *pol* loads [37,86,87,59], which are associated with *C. pecorum* infection [37,31,32,33]. It is possible in early stages of active disease or co-infection that genes involved in cell cycle, innate immunity, adaptive immune responses, and hormone regulation increase KoRV transcription, perhaps leading to more integrations [88]. Over time, increases in KoRV integrations would also increase proviral and viral DNA loads, which are often associated to advanced and chronic disease [89–92], In this study, KoRV proviral loads were more associated to reproductive disease in females. While it is unclear whether reproductive pathology occurs as part of acute or chronic chlamydiosis, this suggests that in these koalas, mechanisms that support reverse-transcription of replication-competent virus are more active. A similar relationship has been observed in koalas with greater KoRV proviral loads and cell-proliferative disorders [43,58]. To unravel the intricacies between KoRV and chlamydiosis pathogenesis, mechanistic studies are required. Given that KoRV is endogenous in northern populations, controlled infection studies cannot be conducted. However, longitudinal monitoring of KoRV integrations in koalas before and during acquisition of natural *C. pecorum* infection, while accounting for co-infections, may determine whether integration-driven promotion of immune dysregulation can explain variations in clinical outcomes [51].

The detection of *C. pecorum* gene transcription in blood was a novel, though not unexpected, finding based on circulation of viable chlamydiae in other species [93] and the detection of chlamydial antigen in the liver and spleen of koalas [94]. Although only seen in eight koalas in this study, the fact that four of these had no evidence of mucosal shedding warrants further investigation as to its significance to pathogenesis and management. We consider these unlikely to be false positives given the absence of detection in 32/33 koalas from a *C. pecorum* free population during assay validation (Fig A in S1 Text). Under current screening and clinical practices, only koalas with clinical signs of disease and/or detection of *C. pecorum* using the *C. pecorum mreC*, *ompB* and 23s rRNA genes at either urogenital or ocular mucosal sites using loop-mediated isothermal amplification (LAMP) and qPCR, target [95] are considered infected and/ or administered antibiotic treatment. The risk posed by circulating *C. pecorum* to individual welfare or transmission to *Chlamydia* free populations, and the ability of currently available treatments to eliminate circulating infections, are unknown. Systemically disseminated *C. pneumoniae* resists elimination by standard anti-chlamydial treatment in humans and is hypothesised to initiate reinfection and generate systemic conditions such as atherosclerosis [96]. It is a reasonable proposition to investigate whether the same is true for *C. pecorum*. As a priority for research and diagnosis, we need to determine whether *C. pecorum* can be detected in blood samples by the more accessible current LAMP and qPCR methods; this would facilitate research to understand the pathogenesis and significance of this finding.

## Conclusion

The high frequencies of co-infections in koalas admitted to rehabilitation and their important association to *C. pecorum* infection and chlamydiosis in this study shifts the classical paradigm of koala chlamydiosis pathogenesis to one which includes multiple co-infection interactions. It highlights the complexity of pathogenesis of chlamydiosis in koalas and that interdisciplinary approaches combining mechanistic in-vitro studies with multivariate analyses incorporating diverse pathogen, environment and host factors are needed to understand these dynamic interactions and improve disease treatment and mitigation strategies.

## Methods

### Ethics statement

Sampling of koalas was conducted under the University of Sydney Animal Ethics Approval Number 2021/1975, NSW NPWS Scientific License SL102379 and Qld NPWS WA0019256.

### Sample Collection

**Fig 5:**
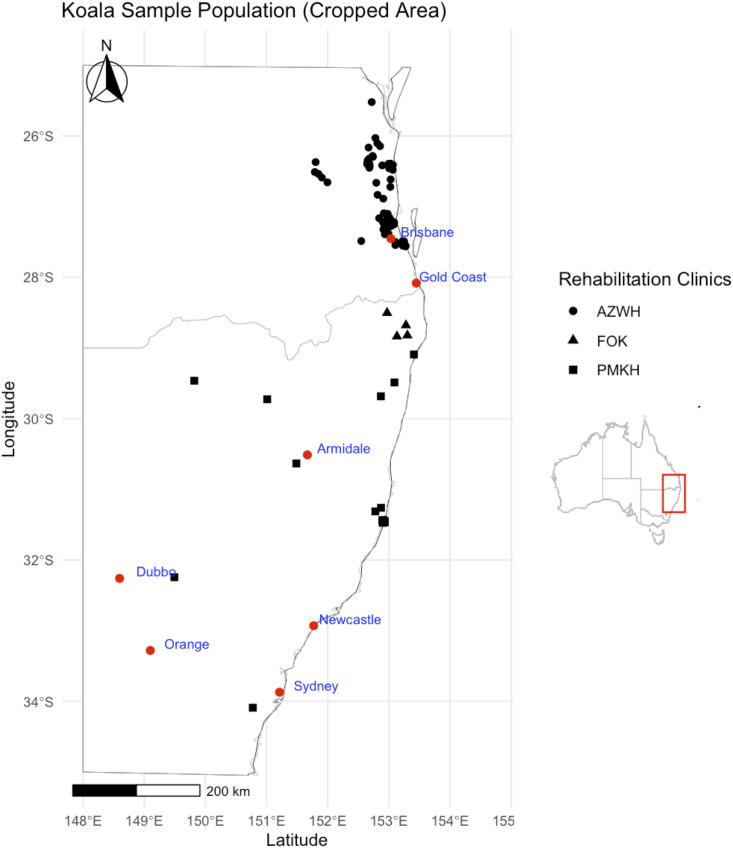
Map of Australian states Queensland and New South Wales (truncated) and the distribution of sampled koalas. Geographical points represent the location (latitude and longitude) from which each individual koala was rescued from before admission to hospital with the shape of points depicting which hospital they were admitted to; Australia Zoo Wildlife Clinic (AZWH, circle), Friends of the Koala (FOK, triangle), and Port Macquarie Koala Hospital (PMKH, square). Red points represent major cities in proximity to koala rescue locations. This map was generated in R using the free vector and raster map data from the Natural Earth public domain package using ne_countries(country = “Australia”) (Natural Earth v4.1.0, March 2018, https://www.naturalearthdata.com/about/terms-of-use/).

Sampling was conducted from September 2021 to April 2022 at three koala care facilities servicing South-east Qld, Northern and Mid-North Coast NSW, and Central NSW (Fig 5). Sampling was completed at Australia Zoo Wildlife Hospital, Port Macquarie Koala Hospital, and Friends of the Koala between September and December 2021, January to April 2022, and February 2022, respectively. All koalas admitted during these periods were considered for inclusion, except cases of extreme trauma where the koalas’ condition or instability under general anaesthetic (GA) could not support sampling, as advised by the veterinarian; or koalas that were dead on arrival. Sampling was performed under GA in accordance with the hospitals’ routine procedures: induction via intramuscular injection of 3 mg/kg alphaxalone (Alfaxan-CD, RTU; Jurox Pty Ltd., Rutherford, Australia) followed by maintenance using 2% isoflurane in 100% oxygen delivered either through face mask or via cuffed endotracheal tube.

Blood (3 mL) was collected from the cephalic or saphenous veins into an EDTA tube (Vacuette Tube, Greiner Bio-One GmBH, Kremsmünster, Austria) and processed into whole blood and buffy coat separations. EDTA whole blood was divided into two 300 µL aliquots, one preserved in 900 µL RNAlater (Qiagen). The remaining EDTA blood was then centrifugated at 1,000 x g for 10 min. From the separated EDTA blood, 250 µL of the buffy coat layer was aliquoted with 750 µL RNAlater (Qiagen) for RNA analysis. All cryogenic vials containing RNAlater (Qiagen) were left at ambient temperature for 24 hours and then stored at -20°C until analysis. All other cryogenic vials were stored at -80°C until analysis. Dry aluminium shaft cotton tipped swabs (Copan Italia, Brasica, Italy) were used to sample the urogenital mucosa (the urogenital sinus in females and the urethra in males), oropharyngeal mucosa, and left and right conjunctivae. Swab tips were cut into separately labelled cryogenic vials (Biologix Grp Ltd, Kansas, United States) and stored at -80°C.

Clinical information included data recorded from visual assessments, ultrasonography, x-ray, haematology and serum biochemistry, urinalysis, paracentesis, point of care *C. pecorum* LAMP assays, laparotomy examinations, faecal wet preparations, blood smears, bone marrow aspirates, and post-mortem assessment. When conducted, all clinic-based loop-mediated isothermal amplification (LAMP) testing of ocular and urogenital swabs was performed to detect *C. pecorum* shedding, according to the protocols described by Hulse et al. [95], with the Genie II (OptiGene, Horsham, South of England, UK). Diagnostic modalities were employed based on the veterinarian’s assessment of each individual case and influenced by facility protocols. Clinical examination data was collated by pooling information recorded by the veterinarian and nurses using the standard hospital recording sheets and a standardised recording sheet designed by the researcher. Some euthanised cases were not assessed by radiography due to severity of trauma, emaciated body condition or acute deterioration in clinical condition. In these cases, necropsies were performed to identify internal abnormalities (N = 18). Necropsies were conducted on site at respective hospitals with the majority completed within 24 hours of euthanasia. Most carcasses were stored at 0 – 4°C until necropsy that same day. Three were examined after 48 hours after storage at 0 – 4°C. All necropsies followed the methods outlined by the Koala Health Hub [97]. Photos were taken to accompany written records of necropsy findings.

### Categorisation of clinical groups

Clinical groups of interest included the presentation of clinical chlamydiosis, untreatable chlamydiosis, and reproductive disease in females, specifically. These were used as dependent binary outcome variables. As outlined in the clinical criteria table (Table A in S1 Text), cases were designated (1) ‘clinical chlamydiosis’ if presenting with wet-bottom (evidence of recent or current incontinence) and/or conjunctivitis (swelling, proliferation and/or inflammation of the conjunctiva + /- cataracts or ocular ulceration), cystitis (thickened bladder walls and inflammation observed on ultrasound using doppler) +/- other urinary tract abnormalities including renal disease (hyperechoic medulla on ultrasound, hydronephrosis, hydroureter), and/or reproductive disease observed on ultrasound (ovarian bursal cyst(s), pyometra, uterine oedema, endometriosis). It should also be noted that koalas with any of these clinical signs may also have presented with other abnormalities such as candidiasis, anaemia, poor body condition, trauma, etc, and that it is possible for unidentified pathogens to have caused clinical signs of disease. If koalas presented with no abnormalities, or with abnormalities but without clinical signs attributable to *C. pecorum* infection, they were classed as (0) ‘no clinical signs of chlamydiosis’. Koalas with clinical chlamydiosis were sub-classified as (1) ‘untreatable chlamydiosis’ if they were euthanised on admission or (0) ‘admitted for chlamydiosis treatment’ to explore the relationships between co-infections and the severity of clinical conditions. Euthanasia was elected on welfare grounds where prognosis was poor due to untreatable/irreparable structural damage to tissues/organs derived from disease and/or trauma, or complex comorbidities in conjunction with emaciation and/or mature age (age > 10 years old). Finally, to determine the importance of co-infections to the prediction of reproductive disease, female koalas were classified as either (1) presenting with evidence of ‘reproductive disease’ as described above, or (0) ‘no evidence of reproductive disease’.

### Pathogen detection techniques

#### Gene transcription.

Of the 115 koalas sampled on admission, 101 had buffy coat samples available for RNA extraction. Adequate buffy coat samples are difficult to collect when the initial blood collection is low in volume or becomes clotted. This is often the case in koalas with clinically severe trauma or disease where dehydration and shock decrease the blood pressure. RNA was extracted from available buffy coat samples stored in RNAlater using the RNeasy Mini Kit (QIAGEN) following the manufacturer’s instructions. The purity and concentration of nucleic acid in extracted RNA samples was determined using a Nanodrop Spectrophotometer ND-1000 (Thermo Fisher Scientific Co., Waltham, MA, USA) followed by specific quantification of RNA and measure of RNA integrity and quality (IQ) using the Qubit RNA HS Assay Kit (Q32852) and Qubit RNA IQ Assay Kit (Q33222) on the benchtop Qubit 4 Fluorometer (Invitrogen, Thermo Fisher Scientific), respectively. The extracted RNA samples were stored at −80 °C until required.

A custom NanoString nCounter plex-set (NanoString Technologies, WA, USA) was designed to quantify transcribed mRNA from a multiplexed set of 72 genes of interest for koala biological and pathological pathways (Table B in S1 Text). While different panels may demonstrate variations in sensitivity and specificity due to probe-specific biological variations, overall the NanoString nCounter platform has demonstrated comparable or improved results compared to RT-qPCR [98,99], microarrays [100], immunohistochemistry and fluorescence in situ hybridisation [98], and RNA-Seq [101,102]. For this study, only pathogen gene targets (14/72) were examined to determine relationships between infection status and clinical outcomes in koalas. Similarly to previous NanoString panel designs for the koala [103,104,105], four housekeeping genes (*GAPDH, ACTB, Stx12, Nckap1l*) were included for normalisation of data [106].

The fourteen infectious agent genes included four KoRV genes targeting three major *env* subtypes (*KoRVAenvRBD, KoRVBenvRBD* and *KoRVDenvRBD),* the immunosuppressive domain *(KoRVenvCKS17),* and the *pol* gene (*KoRVpol)* [105]. For *C. pecorum,* the single-copy conserved hypothetical protein *CpecG_0573* was included as a general *C. pecorum* species detection target [107,108], the chaperonin GroEL gene encoding the *C. pecorum* heat-shock protein 60 *(Cpec_hsp60)* [109], *C. pecorum* strain L17 major outer membrane protein (*ompA*) gene [110,105], and *C. pecorum* strain L1 plasmid pCpecL1 (*Pgp3)* gene was included [111]. For PhaHV detection, the PhaHV-1 [52] and PhaHV-2 [53] specific DNA dependent DNA polymerase gene (*dpol)* was included. Finally, for trypanosome detection the species-specific 18s rRNA regions were targeted for *Trypanosome copemani* [112], *T. gilletti* [112], and *T. irwini* [113]. Blasting of the *C. pecorum* gene probes confirmed specificity to the *C. pecorum* species. Given that buffy coat samples were used, detection of transcription for each infectious agent gene is hereafter referred to as ‘circulating infection’ of each target.

A total concentration of 50 – 100 ng in a total volume of 7–10 µL of eluted RNA was prepared using RNA/DNA free water to adjust concentrations. Additional quality control and sample normalisation was completed by Ramaciotti Centre for Genomics, UNSW, Sydney, Australia preceding mRNA analysis and transcript counting which was performed by the same institute according to the manufacturers protocol (NanoString Technologies, WA, USA). Briefly, this process included mRNA hybridisation with both reporter and capture probes according to the nCounter XT CodeSet Gene Expression Assays Protocol (NanoString Technologies, WA, USA). A NanoString nCounter FLEX Analysis System (NanoString Technologies, WA, USA) was then used as per the manufacturer’s recommendations for purification and transcript counting. Final transcript counts were determined using a Digital Analyzer (NanoString Technologies, WA, USA).

Raw data was analysed using nSolver 4.0 Analysis Software (NanoString Technologies, WA, USA). Briefly, Reporter Code Count (RCC) files containing barcode counts from each gene and control within each lane in the CodeSet and Reporter Library Files (RLF) including instrument and gene probe information were loaded into nSolver. Only samples with housekeeping gene transcription levels exceeding 50 counts were considered for analysis. Using this criterion, 7 samples were omitted from the analysis.

While a trypanosome, PhaHV-1 or PhaHV-2, or KoRV free population was not available to validate the detection threshold for gene mRNA counts, data from a *C. pecorum* free population was. We examined the raw counts for *C. pecorum* gene targets, *Cpec_hsp60* and *CpecG_0573* in a small sample set acquired from a known “Chlamydia free” wild koala population in Campbelltown, NSW, Australia (N = 33). For this Campbelltown sample set, samples were obtained, processed, stored, and extracted utilising the same methods reported above. A box-plot scatter plots visualisation of this data is presented in the supporting information (Fig A and B in S1 Text).

Manufacturer recommendations [114,115] indicated transcription counts below 20 cannot be discerned from background noise and, for *C. pecorum*, this was verified by validation against *C. pecorum* free animals. Therefore any samples with raw expression levels below this threshold were considered negative and those above considered positive for the associated infectious agent. The distribution of raw counts above and below the threshold, and the resulting number of samples classified as ‘positive’ for gene transcription is presented in Fig B in S1 Text. Quantification of gene transcription was conducted in samples that were considered positive for detection of an infectious agent. In these samples, counts were normalised against housekeeping genes (*GAPDH, ACTB, Stx12 & Nckap1l*) to account for differences in sample content.

#### Mucosal *C. pecorum* DNA qPCR.

DNA was extracted from urogenital (UGT) and ocular swabs using the MagMAX CORE Nucleic Acid Purification Kit (Thermo Fisher cat# A32702; ThermoFisher Scientific, Waltham, MA, USA) with modifications to the manufacturer’s instructions. Swab samples were shaved into a 1.5 mL tube containing 350 µL of MagMAX CORE Lysis Solution and 10 µL of Proteinase K and incubated for 1 hour at 56°C. The lysate was then added to a 96DW-plate containing 350 µL of MagMAX CORE Binding Solution and 20 µL of MagMAX CORE Magnetic Beads, then immediately processed on a KingFisher Flex automated extraction instrument using the MagMax_CORE_Flex protocol. Each extraction batch contained a sterile unused swab as an extraction blank. DNA was eluted to a final volume of 100 µL and stored at -80°C until analysis.

Extracts were assessed in a multiplex real-time qPCR using a CFX96 Touch Real-Time PCR Detection System with the corresponding CFX Maestro software (Bio-Rad, Australia). Briefly, this PCR included a *Chlamydia* genus (23S) and species (*C. pecorum*) *ompB* gene primer set as well as a sample quality control that quantified host DNA by amplification of the koala *β-actin* gene. Detailed information on the primer set adapted from [116] is described in Table C in S1 Text. A total PCR reaction volume of 20 µL consisted of 400 nM of each primer, 200 nM of each probe, 10 µL of SensiFAST Probe No-ROX (Bioline cat# BIO-86005), 4.4 µL dH_2_O and 2 µL of DNA template. Samples were analysed in duplicates and a negative control (no template control; dH_2_O) was included. A pUCIDT-AMP vector (Integrated DNA Technologies, USA) containing the 3 target regions and flanking sequences (*β-actin*, *C. pecorum* and *Chlamydia* genus) was used as a synthetic positive control to generate a quantification standard curve at 10-fold dilutions, ranging from 10^3^ to 10^7^ copies. PCR plates were prepared manually or using a Myra Liquid Handling System (Bio Molecular Systems). qPCR conditions consisted of an initial 3-min denaturation at 95°C (1 cycle) followed by 40 cycles of a 10 s denaturation at 95°C and a 40 s annealing at 58°C.

As a quality control, samples that repeatedly failed to amplify *β-actin* were not included in further analysis (ocular samples, N = 6; UGT samples, N = 7). Samples were considered positive if amplification of *β-*actin and either *C. pecorum ompB or* 23s genus, was achieved in both duplicates. Any sample with discordant results between duplicates was retested and samples that failed to amplify were re-run at 1:10 dilution to dilute inhibitors. LOD was determined using probit regression analysis for this assay and was found to be 86 copies of *C. pecorum* per reaction (95%CI) [117]. Quantitative results were reported as *β-*actin normalised *C. pecorum* counts by dividing *ompB* gene counts by *β-*actin gene counts. qPCR efficiencies were between 89–100% and intra-assay variation below 5% for all genes.

#### PhaHV-1 & -2 DNA qPCR.

DNA was extracted from oropharyngeal swabs using the same methods described for urogenital and ocular swabs above. Oropharyngeal swab extracts were analysed using the same PhaHV-1 *dpol* and PhaHV-2 *dpol* DNA qPCR design and method described by [118] and [119], respectively. Koala *β-actin* was included as a DNA quality and quantity control [116]. The primers listed in Table C in S1 Text were used at a concentration of 250 nM in a total reaction volume of 20 µL using 2 µL DNA template and SsoAdvanced Universal SYBR Green Supermix (Bio-Rad, Australia #1725270). A pMG-Amp vector (Macrogen, South Korea) synthetic plasmid (positive control) containing the target region and flanking sequence (PhaHV-1: 166 bp, PhaHV-2: 87 bp) was used to generate a standard curve at 10-fold dilutions to quantify viral loads (ranging from 10^3^ to 10^7^ copies/µL). qPCR conditions consisted of an initial 3-min denaturation at 95°C (1 cycle) followed by 40 cycles of a 10 s denaturation at 95°C, and then a 30 s annealing/extension at 56°C. Finally, a melt curve at 56–95°C for PhaHV-1 and 65 – 90°C for PhaHV-2 at 0.5°C increments was produced. A sample was considered positive if both duplicates amplified *β-actin* and produced a melt curve at 81 – 81.5°C for PhaHV-1 and/or at 87 – 87.5°C for PhaHV-2. Any sample with discordant results between duplicates was retested and samples that failed to amplify were re-run at 1:10 dilution to dilute potential inhibitors. Samples which failed to amplify koala *β-actin* were excluded from further analysis (PhaHV-1, N = 4; PhaHV-2, N = 1). For quantitative analysis, the limit of quantification (95%CI) of PhaHV-1 and PhaHV-2 was 12 copies and 133 copies per reaction, respectively. No sample positive for PhaHV-1 were below the LOQ. Although 10/24 PhaHV-2 positive samples were below the LOQ, these values represented 1.6% of the total distribution and so were included in quantitative analysis as the error was considered insignificant. qPCR efficiencies ranged between 90.2–103.9% for PhaHV-1 and 90.2-96.7% for PhaHV-2 and the inter-assay variation was less than 5% for both targets.

#### KoRV *pol* (cDNA) and proviral (DNA) qPCR.

For KoRV *pol* expression, 16 µL of extracted buffy coat RNA, extracted using methods previously described for NanoString, were DNAse treated as neat samples using the DNase I, RNase-free (1U/ µL) kit #EN0521 (Thermo Scientific) alongside a blank control (dH_2_O) following the manufacturer’s instructions. A final volume of 20 µL DNAse treated samples underwent cDNA synthesis using RevertAid First Strand cDNA Synthesis Kit (#K1622, ThermoFischer) following the manufacturer’s instructions. Cycling was completed using a T100 Thermal Cycle (Bio-Rad) and consisted of 5 min at 25°C, followed by 42°C for 1 hour, then 70°C for 5 min. RT positive and RT negative cDNA products, and DNAse and cDNA blank controls were then assessed in a KoRV *pol* quantitative PCR (RT-qPCR).

For KoRV proviral *pol,* DNA was extracted from EDTA whole blood samples using a MagMAX CORE Nucleic Acid Purification Kit (Thermo Fisher cat# A32702; Thermo Fisher Scientific, Waltham, MA, USA). Briefly, 200 μL of EDTA blood was added into a 1.5 mL tube containing 350 μL of MagMAX CORE Lysis Solution and 10 μL of Proteinase K and incubated at 56 °C for 10 min. The lysate was then added to a 96DW-plate containing 350 μL of MagMAX CORE Binding Solution and 20 μL of MagMAX CORE Magnetic Beads, then immediately processed on a KingFisher Flex automated extraction instrument, using the MagMax_Core_Flex protocol. DNA was eluted to a final volume of 100 μL. Each extraction batch contained one no-DNA blank (sterile PBS) to control for potential contamination. DNA extracts were then diluted 1:10 using dH_2_O for qPCR.

Both diluted DNA and neat cDNA were assessed using the same KoRV *pol* qPCR protocol. PCR plates were prepared manually and using a Myra Liquid Handling System (Bio Molecular Systems). Separate master mixes were generated for KoRV *pol* gene detection and koala *β-actin* reference gene detection and applied to independent wells so that DNA (for whole blood extracts) and cDNA (for buffy coat extracts) quality and quantity could be assessed in real-time alongside KoRV *pol*. Primer and probe sets used are described in Table C in S1 Text. For each KoRV *pol* reaction, a total volume of 20 μL comprised of 0.6 μL of primer (10 μM), 0.2 μL of probe (10 μM), 10 μL of SensiFAST Probe No-ROX (Bioline cat# BIO-86005), 6.6 µL dH_2_O and 2 μL of template. Each 20 μL *β-actin* reaction comprised of 0.8 μL of each primer (10 µM), 0.4 μL of the probe (10 µM), 10 μL of SensiFAST Probe No-ROX (Bioline cat# BIO-86005), 6 μL dH_2_O and 2 μL of template.

Samples were run in duplicates alongside a serial dilution of a positive standard (synthetic KoRV *pol* positive control) and an NTC (no template control; dH_2_O). qPCR conditions consisted of an initial 3 min denaturation at 95°C (1 cycle) followed by 40 cycles of a 10 s denaturation at 95°C and a 40 s annealing at 60°C. The limit of quantification for this assay was determined to be 21 copies per reaction and no samples were excluded. For KoRV *pol* qPCR using cDNA, only copy counts from samples with at least a 10 cycle (Ct) difference between the respective RT- and RT+ were retained for further analysis [120]. Circulating KoRV *pol* quantities are reported as *β*-actin normalised KoRV *pol* copies per mL of template by taking the ratio of KoRV *pol* counts per μL to koala *β*-actin counts per μL x 1000. qPCR efficiencies for the KoRV *pol* gene ranged between 90.6–100% for analysis of DNA samples and 87.2-103% for cDNA samples and the inter-assay variation being less than 5% and less than 6%, respectively.

### Statistical Analysis

The distribution of age in the population was assessed using histograms and Shapiro-Wilk normality tests. Due to a non-normal distribution, age was reformed as a 3 levelled factor: young adult (1.5-3 years old), adult (>3–9 years old), and aged (>9–12 years old). The proportions of male and female koalas were compared using chi-squared tests of independence and were considered equally distributed between all infectious agent groups but showed significant differences in select clinical groups. Co-infection combinations are presented in a Venn diagram.

For Generalized Linear Models (GLM), all non-ubiquitously detected markers were transformed into binary variables (detected (1), not detected (0)) to improve the assessable sample sizes. To ensure meaningful contributions to model estimations by avoiding low variation and high data skewness, only markers with a minimum of 10 events were included in GLM analysis as independent variables [121,122,123]. Hence, circulating *C. pecorum,* PhaHV-1, PhaHV-2, and *T. gilletti* transcription status were excluded. Correlations between the five ubiquitous KoRV markers (KoRV *pol* cDNA/mL, KoRV *pol* DNA/mL, KoRV *env* A mRNA, KoRV *env* D mRNA, KoRV *env* CKS17 mRNA) were assessed using Spearman’s Rank correlations, firstly without considering KoRV *env* B (N = 95) and then in KoRV *env* B positive koalas (N = 29). In the cases of strong and significant co-correlations (r > 0.7, p < 0.05), the parameter with the greatest sample size was retained for further analysis (KoRV *pol* cDNA/mL). Residual plots were constructed, and Shapiro Wilk tests performed to check for normality of continuous variables. Log_10_ transformation was applied to non-normal variables.

Four separate GLMs were conducted to test the effect of co-infection variables on (1) mucosal *C. pecorum* detection (N = 87), (2) the presentation of clinical chlamydiosis (N = 87), (3) untreatable chlamydiosis (N = 48), and (4) reproductive disease in females (N = 50). These are referred to as “global models (1-4)”. In each global model, age group (young adult, adult, & aged), mucosal PhaHV-1, PhaHV-2, and circulating *T. copemani, T. irwini* and KoRV-B detection were used as independent factor variables and KoRV *pol* transcription and proviral loads as independent continuous variables. Mucosal *C. pecorum* detection was included as an independent factor variable in global models 2-4. Sex (male = 0 or female = 1), was included as an independent factor variable in global models 1-3. Observations with missing data for any of the assessed variables were removed as required for GLMs. To the best models and the relative importance of each independent variable on the outcome, global model coefficients were standardised and model averaging was undertaken using the MuMIn package [124]. Comparison of the corrected Akaike’s Information Criterion (AIC_c_) estimations between models as well as two associated measures of model fit, delta AIC_c_ (Δ_i_) and Akaike weights (*w*_*i*_), was used to assess the ”best” fitting models. Akaike weights (*w*_*i*_) are a measure of relative strength of evidence for models [125]. Only models returning Δ_i_ < 2 were considered for model averaging. Precision of model-averaged estimates, also termed the unconditional SE, and unconditional 95% confidence intervals were calculated. All statistical analysis were performed using R Statistical Environment (Version 2022.12.0 + 353) (R Development Core [126] and statistical significance threshold of p-value < 0.05 was applied to all tests conducted. A network plot was generated to summarise the relative importance of predictors with RI > 0.5 and the direction of the interaction between predictors and outcome variables. The thickness of arrows between predictors and outcome variables reflects the magnitude of RI (0.5-1) and the colour indicates a positive (black) or negative (red) interaction.

## Supporting information

S1 Text Supporting Information for the Manuscript. (DOCX)

S1 Data Full Data. (CSV)
