# Supplementary material for: The relationships of viral and protozoal co-infections to Chlamydia pecorum infection and chlamydiosis outcomes in northern koalas (Phascolarctos cinereus)
Source: PLoS Pathog. 2025 Nov 24;21(11):e1013632. doi: 10.1371/journal.ppat.1013632 (PMC12643275; doi:10.1371/journal.ppat.1013632)
Supplement: S1 Text — (DOCX) [file ppat.1013632.s001.docx]

**Supporting Information for the Manuscript:** The relationships of viral and protozoal co-infections to *Chlamydia pecorum* infection and chlamydiosis outcomes in northern koalas *(Phascolarctos cinereus).*

Yasmine S.S. Muir^1^, Belinda R. Wright^1^, Andrea Casteriano^1^, Mathew S. Crowther^2^, Mark B Krockenberger^1^, Amber Gillett^3^, Damien P. Higgins^1*^

1. Sydney School of Veterinary Science, Faculty of Science, University of Sydney, Camperdown, New South Wales, Australia.
2. School of Life and Environmental Sciences, Faculty of Science, University of Sydney, Camperdown, New South Wales, Australia.
3. Australia Zoo Wildlife Hospital, Beerwah, Queensland, Australia.

*damien.higgins@sydney.edu.au

*Classification of pathogen ‘detection’ using NanoString:*

Samples taken from koalas originating from a *Chlamydia*-free population (n = 33) did not demonstrate raw counts above the limit of detection (LOD) threshold (20 mRNA counts) for *Cpec_Hsp60* mRNA counts (Fig A). One koala demonstrated *CpecG_0573* mRNA counts above the LOD (Fig A).


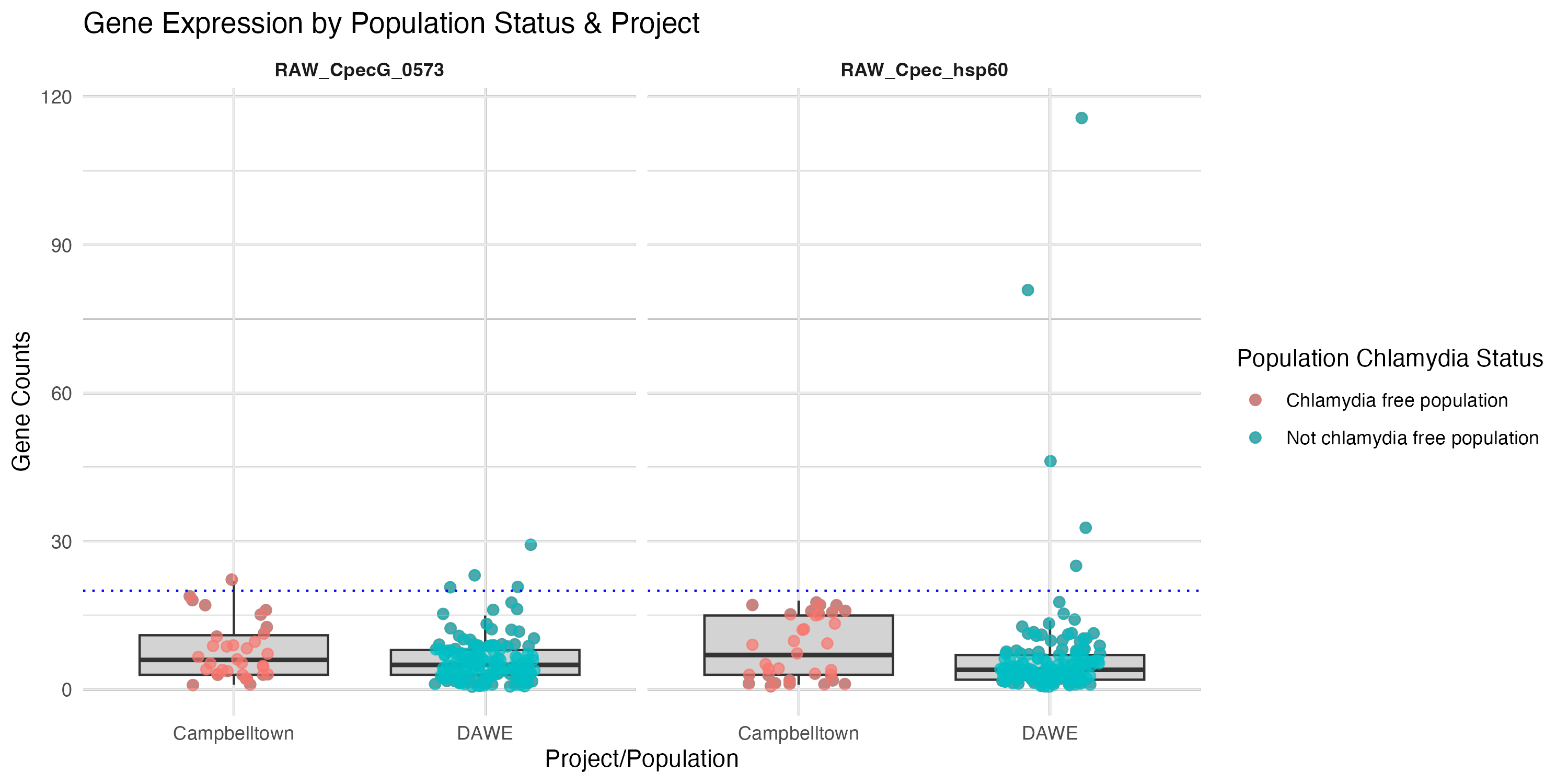


***Fig A: Raw mRNA counts for* C. pecorum *gene targets quantified using NanoString nCounter between two datasets obtained from Chlamydia-free and Chlamydia-affected koala populations.***

*Two scatter-box-plots present the raw mRNA counts for C. pecorum gene targets: Cpec_hsp60 & CpecG_0573. Counts are separated by dataset and coloured by Chlamydia status: Campbelltown – a chlamydia free koala population (pink), and this study – sample populations of koalas from Chlamydia-affected areas analysed (blue).*

Using raw counts, most infectious agent genes were detected above the LOD (20 counts) in at least one sample (Fig B). As expected due to their high transcription loads in northern koalas (1-4), all raw counts were above the LOD for several KoRV genes: *KoRVAenvRBD, KoRVDenvRBD, KoRVenvCKS17,* and *KoRVpol.* Therefore, counts for these four genes were normalised for further analysis as continuous variables.


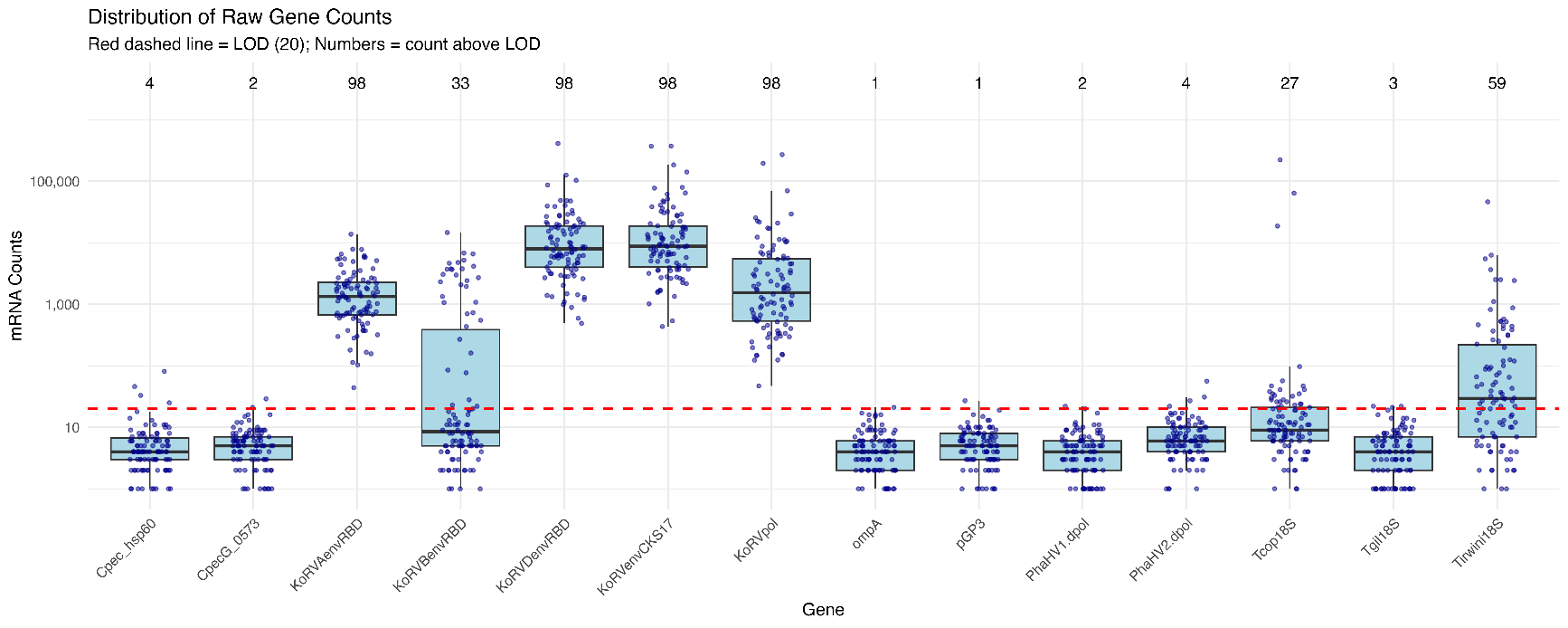
***Fig B: Raw mRNA counts according to gene and the number of cases above the LOD.***

*Box-plots with scatter points are displayed demonstrating the distribution of raw counts according to gene. The limit of detection (LOD) for NanoString is plotted as a red dotted line at 20 counts on the y-axis to demonstrate counts that fall above or below the threshold. The number of cases with counts above the LOD, which were classified as positive for gene transcription detection are indicated above each box-plot. In total, 98 koalas had analysable data from NanoString.*

**Table A: Inclusion criteria for allocation of koala syndromes on admission**

| **Clinical Criteria** | | **Analytical Groups** | | |
| --- | --- | --- | --- | --- |
| **Admission Syndromes** | **Clinical Signs** | **Chlamydiosis** | **Euthanasia?** | **Female Reproductive disease** |
| Chlamydiosis | 1. Reproductive Disease (incl. ovarian bursal cyst(s), pyometra, uterine oedema, and endometriosis) | Chlamydiosis | Yes = Untreatable Chlamydiosis  No =  Treatable Chlamydiosis | Female = Yes |
|  | 1. Cystitis |  |  | Female = No |
|  | 1. Renal Disease (incl. hydronephrosis, hydroureter) |  |  |  |
|  | 1. Conjunctivitis |  |  |  |
|  | +/- Wet bottom (incl. rump ulceration) |  |  |  |
|  | +/- Candidiasis |  |  |  |
|  | +/- C. pecorum LAMP positive |  |  |  |
|  | +/- Fibrotic (irreversible) change to urogenital structures |  |  |  |
|  | +/- Infection associated anaemia |  |  |  |
|  | +/- Poor body condition / emaciation |  |  |  |
|  | +/- Cataracts  +/- Other (Pulmonary infection, growths, alopecia, pustules) |  |  |  |
| Chlamydiosis & Trauma | Any one or combination of clinical signs listed in ‘Chlamydiosis’ with at least one or more clinical signs listed in ‘Trauma’ |  |  |  |
| Trauma | 1. Superficial wounds | No clinical signs of chlamydiosis | NA |  |
|  | 1. Abdominal and/or visceral trauma (incl. haemoabdomen, peritonitis, metabolic alkalosis) |  |  |  |
|  | 1. Single or multiple fractures (incl. comminated, compound, and pathogenic fractures) |  |  |  |
|  | 1. Wound associated septicaemia |  |  |  |
|  | 1. Vestibular & neurological trauma |  |  |  |
|  | 1. Trauma associated anaemia |  |  |  |
| Other Disease | 1. Hepatitis |  |  |  |
|  | 1. Myelodysplasia |  |  |  |
|  | 1. Mammary adenocarcinoma |  |  |  |
|  | 1. Pulmonary Disease |  |  |  |
|  | 1. Congenital renal disease +/- elevated SDMA |  |  |  |
|  | 1. Congenital enophthalmos & Horners syndrome |  |  |  |
|  | 1. Emaciation without evidence of chlamydiosis |  |  |  |
|  | 1. Dysbiosis / Caecal Dysbiosis Typhlocolitis Syndrome |  |  |  |
| No Disease | 1. Nothing abnormal detected using any of the following diagnostic tools; visual assessment, ultrasonography, C. pecorum LAMP negative |  |  |  |
|  | 1. Mended musculoskeletal abnormalities |  |  |  |

**Table B: NanoString Pathogen and House-Keeping Gene Target Summary**

| **Gene** | **Full Name** | **Accession** | **Position** | **Target Sequence** |
| --- | --- | --- | --- | --- |
| PhaHV1 dpol | Herpesvirus 1 dpol | JN585829.1 | 71-170 | GCTCCAAGGCCTTCATGGAATCCCTATCCACCTCAGATCTAGAATCAATAGTGGGACACACAGTCCCAAGCGAAAAGGACTCATCCCTTCGTGTCATCTA |
| PhaHV2 dpol | Herpesvirus 2 dpol | JQ996387.1 | 108-207 | TCGCTCCTGTCCAAGCTCCTGACCACGTGGCTTGCCCGGCGCAAGGAGATCCGACGCCAACACGCGGCCTGCAACGATCCAGCTCTGCGCACTATTCTGG |
| CpecG_0573 | Cpecorum MC/Marsbar strain | NZ_CM002310.1 | 209-308 | TGGGAGTCTTCCTTACAACAGCCCTGCTTACGCTTTTTTGCCTCTGTGCCATCTTGAAGCCCAAAGCAATCTCAGGTAAGTCTGTAACCAAACCAGAGGT |
| pGP3 | Cpecorum L1 strain | KT223773.1 | 4625-4724 | CAATATTCGTTTGGGACAAATGACCAATGAATTGCAAGACAAGCAAATTATAATAGGCACACCAACTACTCCTACTGTAACAAGCCTCTCTTCTTCTAAT |
| ompA | Cpecorum L17 strain ompA | GQ228181.1 | 19-118 | TCGGCGTTTTTATCCGCCGCATTTTTTGCTGGTGACGCCTCCTTACACGCTTTGCCTGTAGGGAACCCAGCAGAGCCAAGTTTATTAATTGATGGGACGA |
| Cpec_hsp60 | CPE1_RS04735, chaperonin GroEL | Cpec_hsp60.1 | 1084-1183 | CCCTGGGGTTCTAGACGAGTAATTGTATCGGCTGGAGAAGAACCATAATAGCCGTGTTCTAACGTACACAAGCGCACCAAAGAACAGAGAGATCGCTTTG |
| KoRVpol | KoRV pol | NC_039228.1 | 2901-3000 | GGAATACCGATTACACGAAAAGCCGGTCCCTCCTTCTATCGACCCGTCATGGCTCCAACTCTTTCCCATGGTTTGGGCCGAGAAGGCAGGTATGGGACTG |
| KoRVAenvRBD | KoRV-A env | AF151794.2 | 6219-6318 | CCCCTGATTCAAACTATGAACATGCTTATAATCAGATCACTTGGGGAACCCTGGGATGTAGCTATCCCCGGGCCAGAACAAGAATTGCTAGGTCCCAGTT |
| KoRVBenvRBD | KoRV-B env | AB822553.1 | 315-414 | GGTGCTTTATAGTCAGGTCGGCAGGGGGTTAGTCAGGCAATCGAGCTATGGAACCTTGGGGTGTCGCTGTCCCCGAGACCGGAACAGACTGGCTCAATCT |
| KoRVDenvRBD | KoRV-D env | AB828004.1 | 121-220 | AACCCTCACCAACCCATGACTCTCACCTGGCAGGTACTGTCCCAGACGGGAAGTGTCGTTTGGGAAAAGAAAGCAGTCGAGCCACCCTGGACGTGGTGGC |
| KoRVenvCKS17 | KoRV env p15E | AF151794.2 | 7503-7602 | TCCAGAATAGGAGAGGCCTTGATCTGCTATTTTTGAAGGAAGGGGGCCTTTGTGCAGCCCTAAAAGAGGAATGCTGTTTCTATGTTGACCACTCAGGCGC |
| Tcop18S | T. copemani 18s rRNA | GU966588.1 | 595-694 | GTTGGTATTTAAAAGTCCATTGGAGATTATGGGGTCGTGTGACAAGCGGTCGGGCGTGTTGCTTTTTTGGTCTTCACGGCCAGGGCGCCGCCCGTCGCCC |
| Tirwini18S | T. irwini 18s rRNA | FJ649479.1 | 539-638 | CAAGTCTGGTGCCAGCACCCGCGGTAATTCCAGCTCCAAAAGCGTATATTAATGCTGTTGCTGTTAAAGGGTTCGTAGTTGAACTGTGGGCCTTCAAGGC |
| Tgil18S | T. giletti 18s rRNA | GU966589.1 | 715-814 | CGTGCACGGTGTGGTGCGAGGCACTTTGTGTGTTCCCGTCACCCACGCGCACGCCTCTTTCGGCTCGCGGCGCCCAGGAATGAAGGAGGGTAGTTCGGGG |
| GAPDH | Glyceraldehyde 3-phosphate dehydrogenase | XM_020994140.1 | 1136-1235 | CACCACTGGGGGAGCCCACATTCCTAACTTAACGTTCCTGTACTGGGGATCTCATGTCCCCATTCGCATCCTTGTCCCAAAGCACCCCTGTAGTCTGGAG |
| ACTB | Beta-actin | XM_021008442.1 | 1805-1904 | TTTACAGTCTCCCTGGGAGTTTTACGAGATTGGTGCCAGTACTTGGGGGAGGGGAGGAGCTTTACCTGTACACTGACTTAAGACCAGTTCAAATAAAAGT |
| Stx12 | Syntaxin 12 | XM_020983277.1 | 674-773 | AGTTGGCCATTACAGAGCAAGACCTTGAACTTATCAAGGAGCGAGAAACTGCGATCAGGCAACTGGAGGCCGACATTTTGGACGTCAATCAGATATTTAA |
| Nckap1l | Nck-associated protein 1-like | XM_020966159.1 | 2922-3021 | CTGCCCATTCCTTATGGGCCCTATTGAGTGCCTGAAGGATTTCGTCACTCCAGATACAGATATAAAGGTGACCATGAGTGTCTTTGAGCTGGCCTCTGCT |

**Table C: qPCR Primer/Probe Set Information for Chlamydia Multiplex qPCR, PhaHV-1 & -2 qPCR, and KoRV *pol* qPCR**

| **qPCR Assay** | **Gene** | **Amplicon size (bp)** | **End** | **Sequence** | **Reference** |
| --- | --- | --- | --- | --- | --- |
| *Chlamydia multiplex Probe qPCR* | *Chlamydia* (23S rRNA) | *137* | Forward | 5′-GCTCACCAATCGAGAATC-3′ | (5) |
|  |  | *137* | Reverse | 5-‘CCAACACTCCTTTCGGTA-3′ |  |
|  |  | *137* | Probe | ROX-CTGAATACTACGCTCTCCTACCGC-BHQ2 |  |
|  | *C. pecorum* (*ompB* gene) | *141* | Forward | 5′−CCAAGCATAATCGTAACAA-3′ |  |
|  |  | *141* | Reverse | 5′-CGAAGCAAGATTCTTGTC-3′ |  |
|  |  | *141* | Probe | FAM-ACTTGTTGGCAATTCTTCTCTTCACA-BHQ1 |  |
|  | Koala *β-actin* mRNA | *145* | Forward | 5′-CTCAGATTATGTTTGAGACCTTC-3′ |  |
|  |  | *145* | Reverse | 5′-CCTTCATAGATGGGCACA-3′ |  |
|  |  | *145* | Probe | HEX-ACCATCACCAGAGTCCATCACAAT-BHQ1 |  |
| *Phascolarctid herpesvirus 1 & 2 SYBR qPCR* | PhaHV-1 *dpol* | *22* | Forward | 5’-GGGAAGAACTATGTTGGAACGC-3’ | (6) |
|  |  | *20* | Reverse | 5’-TGAGTCCTTTTCGCTTGGGA-3’ |  |
|  | PhaHV-2 *dpol* | *20* | Forward | 5’-GGTGACGTGCAATTCAGTGT-3’ | (7, 8) |
|  |  | *20* | Reverse | 5’-TTTCGAGCATCATGCGTCCT-3’ |  |
|  | Koala *β-actin* mRNA | *145* | Forward | 5′-CTCAGATTATGTTTGAGACCTTC-3′ | (5) |
|  |  | *145* | Reverse | 5′-CCTTCATAGATGGGCACA-3′ |  |
| *KoRV pol Probe qPCR* | KoRV *pol* | *110* | Forward | 5’-TTGGAGGAGGAATACCGATTACAC-3’ | (5, 9) |
|  |  | *110* | Reverse | 5’-GCCAGTCCCATACCTGCCTT-3’ |  |
|  |  | *110* | Probe | FAM-TCGACCCGTCATGGC-BHQ1 |  |
|  | Koala *β-actin* mRNA | *145* | Forward | 5’-CTCAGATTATGTTTGAGACCTTC -3’ | (5) |
|  |  | *145* | Reverse | 5’-CCTTCATAGATGGGCACA-3’ |  |
|  |  | *145* | Probe | HEX-ACCATCACCAGAGTCCATCACAAT-BHQ1 |  |

**References**:

1. Tarlinton RE, Legione AR, Sarker N, Fabijan J, Meers J, McMichael L, et al. Differential and defective transcription of koala retrovirus indicates the complexity of host and virus evolution. J Gen Virol. 2022;103(6).

2. Sarker N, Fabijan J, Seddon J, Tarlinton R, Owen H, Simmons G, et al. Genetic diversity of Koala retrovirus env gene subtypes: insights into northern and southern koala populations. J Gen Virol. 2019;100(9):1328-39.

3. Sarker N, Fabijan J, Owen H, Seddon J, Simmons G, Speight N, et al. Koala retrovirus viral load and disease burden in distinct northern and southern koala populations. Sci Rep. 2020;10(1):263.

4. Blyton MDJ, Young PR, Moore BD, Chappell KJ. Geographic patterns of koala retrovirus genetic diversity, endogenization, and subtype distributions. Proc Natl Acad Sci U S A. 2022;119(33):e2122680119.

5. Hulse LS, Hickey D, Mitchell JM, Beagley KW, Ellis W, Johnston SD. Development and application of two multiplex real-time PCR assays for detection and speciation of bacterial pathogens in the koala. J Vet Diagn Invest. 2018;30(4):523-9.

6. Wright BR, Jelocnik M, Casteriano A, Muir YSS, Legione AR, Vaz PK, et al. Development of diagnostic and point of care assays for a gammaherpesvirus infecting koalas. PLoS One. 2023;18(6):e0286407.

7. Kasimov V, Stephenson T, Speight N, Chaber AL, Boardman W, Easther R, et al. Identification and Prevalence of Phascolarctid Gammaherpesvirus Types 1 and 2 in South Australian Koala Populations. Viruses. 2020;12(9).

8. Church C, Casteriano A, Muir YSS, Krockenberger MB, Vaz PK, Higgins DP, et al. New insights into the range and transmission dynamics of a koala gammaherpesvirus, phascolarctid gammaherpesvirus 2. Sci Rep. 2025;In press.

9. Tarlinton R, Meers J, Hanger J, Young P. Real-time reverse transcriptase PCR for the endogenous koala retrovirus reveals an association between plasma viral load and neoplastic disease in koalas. J Gen Virol. 2005;86(Pt 3):783-7.
